# Precision, time, and cost: a comparison of three sampling designs in an emergency setting

**DOI:** 10.1186/1742-7622-5-6

**Published:** 2008-05-02

**Authors:** Megan Deitchler, Hedwig Deconinck, Gilles Bergeron

**Affiliations:** 1 Food and Nutrition Technical Assistance (FANTA) Project, Academy for Educational Development (AED), 1825 Connecticut Ave. NW, Washington, DC, 20009, USA; 2 Formerly with Children in Emergencies and Crisis (CEC), Save the Children (SC)/US, Suite 500, 2000 M St. NW, Washington, DC, 20036, USA

## Abstract

The conventional method to collect data on the health, nutrition, and food security status of a population affected by an emergency is a 30 × 30 cluster survey. This sampling method can be time and resource intensive and, accordingly, may not be the most appropriate one when data are needed rapidly for decision making. In this study, we compare the precision, time and cost of the 30 × 30 cluster survey with two alternative sampling designs: a 33 × 6 cluster design (33 clusters, 6 observations per cluster) and a 67 × 3 cluster design (67 clusters, 3 observations per cluster). Data for each sampling design were collected concurrently in West Darfur, Sudan in September-October 2005 in an emergency setting. Results of the study show the 30 × 30 design to provide more precise results (i.e. narrower 95% confidence intervals) than the 33 × 6 and 67 × 3 design for most child-level indicators. Exceptions are indicators of immunization and vitamin A capsule supplementation coverage which show a high intra-cluster correlation. Although the 33 × 6 and 67 × 3 designs provide wider confidence intervals than the 30 × 30 design for child anthropometric indicators, the 33 × 6 and 67 × 3 designs provide the opportunity to conduct a LQAS hypothesis test to detect whether or not a critical threshold of global acute malnutrition prevalence has been exceeded, whereas the 30 × 30 design does not. For the household-level indicators tested in this study, the 67 × 3 design provides the most precise results. However, our results show that neither the 33 × 6 nor the 67 × 3 design are appropriate for assessing indicators of mortality. In this field application, data collection for the 33 × 6 and 67 × 3 designs required substantially less time and cost than that required for the 30 × 30 design. The findings of this study suggest the 33 × 6 and 67 × 3 designs can provide useful time- and resource-saving alternatives to the 30 × 30 method of data collection in emergency settings.

## 1. Introduction

### 1.1. Background

#### 1.1.1. Data collection in emergency settings

Appropriate response to a nutritional emergency requires reliable and timely data about the health, nutrition, and food security status of the affected population. The assessment method commonly used in emergency settings is a 30 × 30 cluster survey [1]-a method that provides statistically reliable results if administered and analyzed correctly, but that can be time-consuming and expensive to administer. Studies have been conducted or are currently underway to investigate alternative methods to collect reliable data in emergency settings [2-4].

Deitchler *et al *[3] recently compared the performance of two alternative sampling designs, a 33 × 6 cluster design (33 clusters, 6 observations per cluster) and a 67 × 3 cluster design (67 clusters, 3 observations per cluster), to a conventional 30 × 30 cluster survey for estimation of child-level indicators. Data for the 33 × 6 design, 67 × 3 design, and a standard 30 × 30 design were collected concurrently in the Siraro *woreda *in Ethiopia during the 2003 nutritional emergency. The study showed encouraging results with respect to the statistical reliability and time savings offered by the 33 × 6 and 67 × 3 designs. However, that study did not sample for each design independently; rather, data were shared among the designs when the same primary sampling unit (PSU) was selected for sampling by multiple designs. The investigators recommended that a second study, using independent samples for each design, be carried out to validate the 33 × 6 and 67 × 3 designs [3]. This paper addresses that recommendation.

In the study described here, we use independent samples, representative of the same area, and collected concurrently, to compare the performance of the 33 × 6, 67 × 3, and 30 × 30 designs. Data were collected in the Administrative Units (AUs) of Fur Baranga and Habila in West Darfur, Sudan in September-October 2005 in order to compare: 1) child- and household-level indicator results (point estimates and 95% confidence intervals (CIs)); 2) crude and under five mortality rates; and 3) the time and cost required for data collection among the designs. A secondary objective of the study was to use data from the 33 × 6 and 67 × 3 designs to conduct a Lot Quality Assurance Sampling (LQAS) hypothesis test to assess whether a critical prevalence of global acute malnutrition (GAM) was exceeded in each AU, and to compare the results among designs.

#### 1.1.2 Background on LQAS

LQAS is a quality assurance method frequently applied in international health [5]. In this context, the approach typically uses cumulative binomial probabilities to assess whether a binary outcome is at or above a critical threshold level [6]. A LQAS hypothesis test can be expressed as:

H_o_: p ≥ p_o _vs. H_a_: p < p_o_

where p is the true prevalence and p_o _is the prevalence level the data are tested against.

Use of the binomial distribution for hypothesis testing usually requires that observations in a sample be randomly and independently selected [7]. For population-based surveys in the international health setting, a Simple Random Sample (SRS) is therefore most often used for selection of the sample to be analyzed by LQAS [8].

The use of LQAS in international health requires familiarity with terms such as 'upper and lower thresholds', 'alpha and beta errors', and 'decision rules'. These terms are well described throughout the LQAS literature [3,5,6]. In a typical application, the investigator determines *a priori *the indicator of interest (e.g. GAM), the upper threshold level (p_o_) against which the data will be tested (e.g. GAM equal to or greater than 10%), the tolerable level of statistical error (i.e. alpha and beta), and the desired precision of the hypothesis test (i.e. the spread between the upper and lower threshold). From these parameters, the sample size and decision rule for the LQAS application are established. As with other forms of statistical analyses, the lower the desired error for the hypothesis test, the larger the sample needed. In addition, the smaller the spread between the upper and lower thresholds, the larger the sample needed.

To conduct a LQAS hypothesis test, the number of observations in the sample with the outcome of interest (e.g. acute malnutrition – defined by wasting (weight-for-height z score < -2 standard deviations) or bipedal edema) is counted and this sum compared against a pre-established decision rule. If the sum is less than or equal to the decision rule, we reject the null hypothesis and conclude that the prevalence of GAM is less than the threshold level tested. If the number of children in the sample with acute malnutrition is greater than the decision rule, we fail to reject the null hypothesis and conclude that the area should benefit from humanitarian assistance appropriate for that threshold level.

The possibility for misclassification is present with LQAS, as with any type of hypothesis test. The alpha error is the type I error, or the probability of rejecting the null hypothesis when it is true. This is often referred to as the "consumer risk" in LQAS nomenclature. The beta error is the type II error, or the probability of failing to reject the null hypothesis when it is false, also referred to as the "provider risk" in LQAS.

#### 1.1.3. LQAS hypothesis test of GAM prevalence with the 33 × 6 and 67 × 3 designs

In 2003, the FANTA project, managed by the Academy for Educational Development, in collaboration with Catholic Relief Services (CRS) and Ohio State University (OSU), began an investigation to determine if LQAS methods (using cumulative binomial probabilities) could be used to rapidly assess the prevalence of GAM in emergency settings [9]. Until that time, LQAS had been less frequently used to assess nutritional status indicators – such as GAM – due, in part, to the higher level of statistical precision, and thus, larger sample size, required for measuring these indicators as opposed to health service provision indicators [5].

In contrast to the small sample size generally used for assessment of service provision indicators [8], the FANTA/CRS/OSU team defined a sample size of n~200 to be necessary for LQAS assessment of GAM prevalence. This sample size allows for assessment of GAM against the 10% and 15% upper thresholds (with lower thresholds of 5% and 10%, respectively), while maintaining a maximum alpha error of 0.10 and beta error of 0.20 [9]. In emergencies, the 10% and 15% GAM threshold levels are often used to determine the scale of humanitarian response warranted and the most appropriate type of nutrition intervention to implement [10]. A LQAS hypothesis test to classify the prevalence of GAM against these thresholds therefore has great utility. Collection of a sample of 200 observations by the SRS method used in standard LQAS applications would, however, be onerous and time consuming; and thus impractical in an emergency setting.

To investigate the validity of using a cluster sampling approach, as opposed to a SRS for LQAS assessment of GAM prevalence, the FANTA/CRS/OSU team conducted a series of computer simulations which showed that the binomial probabilities for the LQAS decision rules were accurate when assessing a prevalence of 10%, 15%, and 20% (using lower thresholds of 5%, 10% and 15%, respectively), with a sample size of n~200 and data collected in clusters of size 2–6 [3,9]. That earlier work provided the theoretical basis for using the 33 × 6 and 67 × 3 designs for LQAS assessment of GAM prevalence.

#### 1.1.4. Field validation sites

Fur Baranga and Habila are two of the three administrative units comprising the Habila locality of West Darfur State in Sudan. At the time of this study, the total population of Fur Baranga was estimated as 41,691, and, of Habila, as 43,112. Save the Children (SC)/US started a comprehensive relief program in West Darfur in April 2004. Data collected for this study were used by SC/US to assess the health, nutrition, and food security status and needs of the population in Fur Baranga and Habila. The highly vulnerable and food insecure situation of these areas, along with the volatile security situation, also provided the field conditions appropriate to validate the use of the 33 × 6 and 67 × 3 designs in an emergency setting.

## 2. Analysis

### 2.1. Methods

#### 2.1.1. Data collection

Data for this study were collected in Fur Baranga from September 30–October 5 and in Habila from October 5–October 9, 2005. Information on the health and nutrition status of children 6–59 months, on key household-level indicators such as access to potable water, and crude and under-five-mortality rate were collected. Working in teams of three, trained interviewers from the SC/US health staff in Sudan and the State Ministry of Health administered the questionnaires using Personal Digital Assistants (PDAs).

In each AU, the 33 × 6 and 67 × 3 designs were administered alongside a conventional 30 × 30 design, using the same questionnaire and independent samples. The sampled clusters (villages) were selected independently for each design using Probability Proportionate to Size (PPS). The spin-the-bottle method [11] was used to randomly select the first household to be sampled within each cluster. Subsequent households in each cluster were selected by sampling the nearest household to the right.

Data collected at each household followed the conventional sampling protocol used in emergencies [12,13]. In households that had no children 6–59 months, only the household questionnaire was completed. If the household had one or more children 6–59 months, a questionnaire was completed for each child 6–59 months living in the household, in addition to the household questionnaire. A cluster was considered complete only when data were collected from the minimum number of households and the minimum number of children for that design (e.g., 30 × 30 design: minimum 30 households, 30 children per cluster). This method of sampling maintains self-weighted samples for child- and household-level indicators, and permits analysis of mortality data using a standard 30 × 30 household survey [2,13].

Teams also collected the following time expenditure and travel data: 1) distance (km) of each travel segment; 2) time to complete each travel segment; and 3) time to locate the first randomly selected household in a cluster. The time to administer a questionnaire and the time to walk from household to household were captured automatically by the PDA application.

#### 2.1.2. Data cleaning

Identical data cleaning procedures were applied to all datasets. Anthropometric data were processed against the NCHS/WHO 1978 child growth references using Epi-Info 6.04. Children with anthropometric data flagged by Epi-Info [14] were removed from analysis of anthropometric indicators unless bipedal edema was indicated, in which case the child was retained for analysis of GAM.

Although the sampling protocol was well implemented, some clusters in the datasets were either over- or undersized. The most common situation was oversized clusters in the child-level datasets. This was an expected result of the sampling protocol, which collected data on all children 6–59 months in a household. In these cases, the excess number of children was randomly selected from that cluster for exclusion from analysis. Undersized clusters occurred less commonly, but affected both the child- and household-level datasets for each design, usually as a result of missing data for certain indicators. Individual datasets were established for each indicator, by design, and by AU, in order to establish the most complete dataset for analysis of each indicator.

Time data were cleaned of outlying values. If the distance for a travel segment was missing, the data were replaced either with the distance recorded by other designs for the same travel route or, if not available, by imputing the average distance of all travel routes within the same design. This latter situation was limited to the 67 × 3 design, and was necessary for ten travel segments required for that design.

#### 2.1.3. Data analysis

Point estimates, 95% CIs, design effects, and intra-cluster correlation results for all designs were derived using Intercooled Stata v 9.2 [15]. For point estimate and design effect (deff) calculations, data were weighted inversely proportional to the achieved cluster sample size. Intra-cluster correlations were approximated using the generalized linear latent and mixed models (gllamm) program in Stata. Confidence intervals accounted for the design effect and were derived using the binomial wald method. To conduct the LQAS hypothesis tests for the 33 × 6 and 67 × 3 designs, the number of children with acute malnutrition were counted and then compared against the decision rule for that threshold level.

Time expenditure data were analyzed by calculating the average length of time required to complete each element of data collection by design and by AU. To control for variability related to individual and/or team factors, the average time required to complete a questionnaire and to walk from household to household was derived using time expenditure data across all designs. Detailed information about the average time required for various components of data collection is shown by design in Tables 1 and 2. The total time required to complete data collection was calculated by applying the average time expenditure measures to the cluster and sample size specifications of each design. The time estimation formula used for each design is summarized below.

**Table 1:** Fur Baranga: average time (in minutes) required to carry-out data collection components, by sampling design

	*Sampling design*		
*Data collection component*	*33 × 6 design*	*67 × 3 design*	*30 × 30 design*

Drive to 1^st ^cluster of day	7.9	8.5	11.3
Locate 1^st ^household in cluster	14.2	12.2	14.6
Walk household to household in cluster	3.0	3.0	3.0
Administer questionnaire (0 children in household)	7.1	7.1	7.1
Administer questionnaire (children in household)	15.1	15.1	15.1
Drive to next cluster of same design	5.8	6.7	27.8
Drive back to base at end of day	6.2	11.4	12.8

**Table 2:** Habila: average time (in minutes) required to carry-out data collection components, by sampling design

	*Sampling design*		
*Data collection component*	*33 × 6 design*	*67 × 3 design*	*30 × 30 design*

Drive to 1^st ^cluster of day	29.6	25.8	21.7
Locate 1^st ^household in cluster	8.3	9.8	15.5
Walk household to household in cluster	2.0	2.0	2.0
Administer questionnaire (0 children in household)	4.8	4.8	4.8
Administer questionnaire (children in household)	10.5	10.5	10.5
Drive to next cluster of same design	2.3	2.0	5.2
Drive back to base at end of day	15.4	28.6	20.8

Total estimated time = A + X (B + C + (D × (E-1))) + F (X-1) + G,

where A = the average time to drive to the first cluster of a work day, B = the average time to locate the first household in a cluster, C = the average time to complete all household and child questionnaires in one cluster, D = the average time to walk between households sampled in a cluster, E = the average number of households to be visited to complete one cluster of data collection, F = the average time to drive between clusters, G = the average time to return to base at the end of the work day, and X = the average number of clusters that could be completed in one day of work.

For the time estimation calculations, we allowed the length of the work day to be determined by the average time required to complete one 30 × 30 cluster (or two 30 × 30 clusters in the case of Habila) in one work day. In other words, we assumed that no 30 × 30 cluster would need to be revisited to complete data collection from a previous work day. These assumptions were made for ease of calculation, though we feel they may be overly optimistic with respect to the 30 × 30 design. Therefore, our time estimations may be conservative, and under-represent the true amount of time savings offered by the 33 × 6 and 67 × 3 designs in Fur Baranga and Habila.

Cost estimations for each design account for the following expenditures: interviewer and driver salaries and per diem, rental vehicles, fuel, and paper and printing. Total staff and vehicle expenses were calculated by multiplying daily costs by the number of days estimated to be required for data collection of each design. Fuel costs were calculated using actual distances (km) traveled by design. This method of analysis was found to be most appropriate but did preclude a cost analysis from being conducted in Habila. Whereas in Fur Baranga vehicle itineraries were assigned by design, the logistic plan in Habila allowed for one vehicle to visit clusters for more than one design per day.

### 2.2. Results

#### 2.2.1. Estimation and precision of child-level indicators

One way to gauge the general comparability of results (without conducting statistical tests and therefore claiming statistical significance) is to assess if there is an overlap of the 95% CIs for an indicator among the three designs. Only the indicator for acute respiratory infection (ARI) in Fur Baranga shows variable results among designs when assessing comparability is this way. For the ARI indicator, the CI of the 33 × 6 design overlaps with the CI of the 67 × 3 and 30 × 30 designs, but the CI of the 67 × 3 design and the CI of the 30 × 30 design do not overlap with each other (Tables 3, 4).

**Table 3:** Fur Baranga: point estimates, 95% CIs, and width of CIs (in ppt) for child-level indicators, by sampling design

*Indicator*	*Sampling design*		
	*33 × 6 design*	*67 × 3 design*	*30 × 30 design*

GAM	8.1 (3.6, 12.5) +/- 4.5	6.5 (3.0, 9.9) +/- 3.5	8.6 (6.5, 10.8) +/- 2.2
Low MUAC (<12.5 cm)	5.6 (1.7, 9.4) +/- 3.9	4.5 (1.7, 7.3) +/- 2.8	3.8 (2.1, 5.5) +/- 1.7
Stunting	33.8 (25.0, 42.6) +/- 8.8	36.6 (29.6, 43.6) +/- 7.0	32.3 (29.9, 34.7) +/- 2.4
Underweight	34.3 (26.7, 42.0) +/- 7.7	34.1 (27.9, 40.3) +/- 6.2	31.6 (27.9, 35.2) +/- 3.7
BCG vaccination	33.8 (24.6, 43.1) +/- 9.3	34.3 (26.1, 42.6) +/- 8.3	45.6 (38.7, 52.5) +/- 6.9
Measles vaccination	41.5 (30.5, 52.4) +/- 11.0	52.2 (42.6, 61.8) +/- 9.6	56.9 (47.0, 66.8) +/- 9.9
VAC supplementation (5.5 month recall)	78.4 (68.5, 88.2) +/- 9.9	81.3 (73.4, 89.3) +/- 8.0	83.5 (72.9, 94.1) +/- 10.6
Diarrhea (2 week recall)	11.6 (7.0, 16.2) +/- 4.6	14.9 (9.4, 20.5) +/- 5.6	9.3 (5.9, 12.6) +/- 3.4
ARI (2 week recall)	10.1 (3.7, 16.5) +/- 6.4	16.7 (11.4, 21.9) +/- 5.3	5.9 (2.7, 9.1) +/- 3.2
Fever (2 week recall)	21.2 (14.1, 28.4) +/- 7.2	17.9 (11.7, 24.1) +/- 6.2	14.6 (11.2, 18.0) +/- 3.4

**Table 4:** Habila: point estimates, 95% CIs, and width of CIs (in ppt) for child-level indicators, by sampling design

*Indicator*	*Sampling design*		
	*33 × 6 design*	*67 × 3 design*	*30 × 30 design*

GAM	4.7 (1.9, 7.4) +/- 2.8	8.0 (4.5, 11.5) +/- 3.5	6.9 (4.8, 9.0) +/- 2.1
Low MUAC (<12.5 cm)	4.0 (0.7, 7.4) +/- 3.4	3.0 (0.7, 5.3) +/- 2.3	3.3 (2.0, 4.7) +/- 1.4
Stunting	26.0 (19.1, 32.8) +/- 6.9	25.4 (19.4, 31.4) +/- 6.0	27.3 (23.8, 30.9) +/- 3.6
Underweight	22.9 (16.8, 29.1) +/- 6.2	27.6 (21.1, 34.1) +/- 6.5	27.4 (24.0, 30.9) +/- 3.5
BCG vaccination	26.8 (16.4, 37.1) +/- 10.4	40.3 (31.6, 49.0) +/- 8.7	35.2 (28.0, 42.5) +/- 7.3
Measles vaccination	57.4 (43.7, 71.1) +/- 13.7	55.0 (45.0, 65.0) +/- 10.0	55.4 (45.3, 65.5) +/- 10.1
VAC supplementation (5.5 month recall)	74.1 (61.7, 86.6) +/- 12.5	69.7 (59.7, 79.6) +/- 10.0	77.7 (67.1, 88.4) +/- 10.7
Diarrhea (2 week recall)	6.6 (2.2, 11.0) +/- 4.4	6.0 (2.3, 9.7) +/- 3.7	10.9 (6.6, 15.2) +/- 4.3
ARI (2 week recall)	6.1 (1.9, 10.2) +/- 4.2	6.0 (2.0, 10.0) +/- 4.0	3.4 (1.9, 5.0) +/- 1.6
Fever (2 week recall)	16.2 (9.8, 22.5) +/- 6.4	21.6 (15.3, 28.0) +/- 6.4	18.4 (14.5, 22.2) +/- 3.9

The results for all child-level indicators are less precise (i.e. wider CIs) for the 33 × 6 design than for the 30 × 30 design – with one exception: the indicator reporting vitamin A capsule (VAC) supplementation coverage in Fur Baranga. In Fur Baranga, the difference in the width of the CIs for the 33 × 6 design compared to the 30 × 30 design ranges from +/- -0.7 (VAC supplementation) to +/- 6.4 percentage points (stunting), with a median difference of +/- 2.4 percentage points. In Habila, the difference in the width of the CIs ranges from +/- 0.1 (diarrhea) to +/- 3.6 (measles vaccination) percentage points, with a median difference of +/- 2.6 percentage points.

The CIs for the 67 × 3 design are also wider than those of the 30 × 30 design for most indicators, but the difference in the width of the CIs is smaller than those between the 33 × 6 and 30 × 30 designs. In Fur Baranga, the difference in precision ranges from +/- -2.6 (VAC supplementation) to +/- 4.6 percentage points (stunting) with a median difference of +/- 1.8 percentage points. In Habila, the range is +/- -0.7 (VAC supplementation) to +/- 3.0 percentage points (underweight) with a median difference of +/- 1.4 percentage points.

#### 2.2.2. LQAS hypothesis test of GAM prevalence

Although a SRS is usually required for LQAS hypothesis testing, earlier simulation studies have shown the 33 × 6 and 67 × 3 designs to have alpha and beta errors equivalent to those that would be incurred for testing GAM thresholds when using a SRS of the same sample size [3,9]. Empirically, the GAM data collected in this study meet that SRS expectation. The 67 × 3 design in both AUs and the 33 × 6 design in Habila show a design effect <1 for GAM. (A SRS is considered to have a design effect = 1). Although the 33 × 6 design in Fur Baranga shows a design effect (1.24) slightly above that of a SRS, we expect this is due to the smaller than expected sample size of the final dataset (n = 192 vs. n = 198)

In this field test, the alpha and beta errors associated with the LQAS hypothesis tests were slightly elevated due to the sample size of the 33 × 6 and 67 × 3 designs being less than the ideal n = 198 and n = 201 after data cleaning (Table 5). In Fur Baranga, the 33 × 6 design found 16 children with acute malnutrition (n = 192) and the 67 × 3 design found 12 (n = 194). Both designs indicate the null hypothesis should be rejected at the 20% and 15% GAM thresholds. We conclude, therefore, that Fur Baranga has a GAM prevalence less than 15%. With a decision rule of 13 for the 10% threshold, the designs provide inconsistent results: whereas the 67 × 3 design indicates the null hypothesis should be rejected, the 33 × 6 design indicates the null hypothesis cannot be rejected (Table 6). Similar results emerged from Habila. The 33 × 6 design found 9 children with acute malnutrition (n = 197) and the 67 × 3 design found 16 (n = 199). Again, both designs indicate the GAM prevalence as less than 15% but provide inconsistent results for the 10% threshold (Table 7).

**Table 5:** Decision rules, alpha and beta errors for the 33 × 6 and 67 × 3 designs for the 10%, 15% and 20% GAM thresholds, assuming full sample size available for analysis

*Sampling design*	*n*	*Decision rule (# of children with acute malnutrition) to reject H_o_*		
		
		*10% GAM threshold*	*15% GAM threshold*	*20% GAM threshold*
33 × 6 design	198	≤ 13 (α = 0.06, β = 0.12)	≤ 23 (α = 0.10, β = 0.18)	≤ 33 (α = 0.13, β = 0.22)
67 × 3 design	201	≤ 13 (α = 0.05, β = 0.13)	≤ 23 (α = 0.09, β = 0.20)	≤ 33 (α = 0.11, β = 0.24)

**Table 6:** Fur Baranga: decision rules, alpha and beta errors for the 33 × 6 and 67 × 3 designs for the 10%, 15% and 20% GAM thresholds, using actual sample size available for analysis

*Sampling design*	*# of children with acute malnutrition in sample*	*n (actual sample size)*	*Decision rule (# of children with acute malnutrition) to reject H_o_*		
			
			*10% GAM threshold*	*15% GAM threshold*	*20% GAM threshold*
33 × 6 design	16	192	≤ 13 (α = 0.07, β = 0.10)	≤ 22* (α = 0.09, β = 0.21)	≤ 32* (α = 0.14, β = 0.22)
67 × 3 design	12	194	≤ 13 (α = 0.07, β = 0.10)	≤ 22* (α = 0.08, β = 0.22)	≤ 32* (α = 0.13, β = 0.24)

* Adjusted decision rule due to reduced sample size

**Table 7:** Habila: decision rules, alpha and beta errors for the 33 × 6 and 67 × 3 designs for the 10%, 15%, and 20% GAM thresholds, using actual sample size available for analysis

*Sampling design*	*# of children with acute malnutrition in sample*	*n (actual sample size)*	*Decision rule (# of children with acute malnutrition) to reject H_o_*		
			
			*10% GAM threshold*	*15% GAM threshold*	*20% GAM threshold*
33 × 6 design	9	197	≤ 13 (α = 0.06, β = 0.11)	≤ 23 (α = 0.11, β = 0.18)	≤ 33 (α = 0.14, β = 0.21)
67 × 3 design	16	199	≤ 13 (α = 0.05, β = 0.12)	≤ 23 (α = 0.10, β = 0.19)	≤ 33 (α = 0.13, β = 0.23)

#### 2.2.3. Estimation and precision of household-level indicators

Notwithstanding the 'Report of a household food shortage' indicator in Fur Baranga, the designs show an overlap of the 95% CI for each household-level indicator tested (Tables 8, 9). On average, the 67 × 3 design provides the most precise results for the household-level indicators. In Fur Baranga, the difference in precision between the 67 × 3 and 30 × 30 designs ranges from +/- -4.3 (access to potable water) to +/- 3.0 percentage points (ownership of bed net) with a median difference of +/- -1.1 percentage points. In Habila, the range is +/- -5.7 (access to potable water) to +/- -1.3 percentage points (ownership of bed net) with a median difference of +/- -2.2 percentage points. The 33 × 6 design provides only slightly wider CIs than the 30 × 30 design, showing a median difference of +/- 1.6 percentage points in Fur Baranga and +/- 0.5 percentage points in Habila.

**Table 8:** Fur Baranga: point estimates, 95% CIs, and width of CIs (in ppt) for household-level indicators, by sampling design

*Indicator*	*Sampling design*		
	*33 × 6 design*	*67 × 3 design*	*30 × 30 design*

Access to potable water	61.1 (46.2, 76.0) +/- 14.9	48.3 (36.8, 59.7) +/- 11.5	49.8 (34.0, 65.6) +/- 15.8
Access to latrine	50.0 (36.9, 63.1) +/- 13.1	46.8 (37.6, 56.0) +/- 9.2	44.6 (33.7, 55.4) +/- 10.9
Ownership of bed net	55.8 (45.1, 66.4) +/- 10.7	53.5 (45.2, 61.7) +/- 8.3	47.8 (42.6, 53.1) +/- 5.3
Food shortage (5.5 month recall)	75.3 (64.7, 85.8) +/- 10.6	51.5 (42.3, 60.7) +/- 9.2	47.8 (38.1, 57.4) +/- 9.7

**Table 9:** Habila: point estimates, 95% CIs, and widths of CIs (in ppt) for household-level indicators, by sampling design

*Indicator*	*Sampling design*		
	*33 × 6 design*	*67 × 3 design*	*30 × 30 design*

Access to potable water	52.0 (34.6, 69.4) +/- 17.4	53.2 (41.2, 65.3) +/- 12.1	57.4 (39.7, 75.2) +/- 17.8
Access to latrine	50.5 (37.4, 63.6) +/- 13.1	50.3 (40.4, 60.1) +/- 9.9	50.8 (39.3, 62.3) +/- 11.5
Ownership of bed net	29.3 (17.1, 41.5) +/- 12.2	36.6 (26.5, 46.7) +/- 10.1	35.1 (23.7, 46.5) +/- 11.4
Food shortage (5.5 month recall)	66.7 (53.7, 79.6) +/- 13.0	62.4 (52.4, 72.5) +/- 10.1	61.7 (48.8, 74.6) +/- 12.9

#### 2.2.4 Estimation and precision of mortality rates

In addition to the above indicators, two indicators of retrospective mortality were tabulated using the household sample of each design and methods recommended by Standardized Monitoring and Assessment of Relief and Transitions (SMART) [12]. Fairly similar point estimate results are shown among the designs in each AU; however, in comparison to the 30 × 30 design, the 33 × 6 and 67 × 3 designs show much wider CIs for these indicators (Tables 10, 11). Due to the low mortality rate and wide confidence intervals, the lower bound of the CIs for the 33 × 6 and 67 × 3 designs extends beyond 0. For the results reported here, the lower bound of the CI results is truncated at 0. Estimates of crude and under five mortality rates are typically measured against the 'alert' thresholds of 1/10,000/day and 2/10,000/day, respectively; and the 'emergency' thresholds of 2/10,000/day and 4/10,000/day, respectively [12,16-18]. Whereas the 30 × 30 design produces clear (non-overlapping) classifications for the thresholds, the CIs for the 33 × 6 and 67 × 3 designs show an overlap across multiple threshold levels.

**Table 10:** Fur Baranga: mortality rate estimates, 95% CIs, and sample size (total person days at risk: PDAR), by sampling design

*Indicator*	*Sampling design*		
	*33 × 6 design*	*67 × 3 design*	*30 × 30 design*

Crude mortality rate*	0.67 (0.00^†^, 1.93) n = 179,498 PDAR	0.10 (0.00^†^, 1.28) n = 193,602 PDAR	0.53 (0.34, 0.72) n = 659,362 PDAR
Under 5 mortality rate*	1.17 (0.00^†^, 4.50) n = 42,804 PDAR	0.23 (0.00^†^, 2.50) n = 43,952 PDAR	0.86 (0.32, 1.40) n = 174,988 PDAR

*Reported as deaths/10,000/day and calculated using a recall period of 164 days for the household census information.

^† ^Lower bound of CI truncated at 0 due to low mortality estimate and wide CI.

**Table 11:** Habila: mortality rate estimates, 95% CIs, and sample size (total person days at risk: PDAR), by sampling design

*Indicator*	*Sampling design*		
	
	*33 × 6 design*	*67 × 3 design*	*30 × 30 design*
Crude mortality rate*	0.23 (0.00^†^, 0.85) n = 132,048 PDAR	0.31 (0.00^†^, 1.83) n = 160,020 PDAR	0.18 (0.05, 0.31) n = 626,640 PDAR
Under 5 mortality rate*	0.27 (0.00^†^, 1.55) n = 37,128 PDAR	0.49 (0.00^†^, 4.25) n = 41,160 PDAR	0.36 (0.05, 0.66) n = 169,260 PDAR

*Reported as deaths/10,000/day and calculated using a recall period of 168 days for the household census information.

^† ^Lower bound of CI truncated at 0 due to low mortality estimate and wide CI.

#### 2.2.5. Time expenditure and cost comparisons

The 33 × 6 and 67 × 3 designs demonstrate substantial time-savings over the 30 × 30 cluster survey. In each AU, our analysis shows that data collection for the 33 × 6 design took about one-quarter and for the 67 × 3 design took about one-third of the time required by the conventional 30 × 30 cluster survey (Tables 12, 13). The cost of the 33 × 6, 67 × 3, and 30 × 30 designs in Fur Baranga is estimated as US$1232, US$1630, and US$4606, respectively. These cost calculations assume the use of paper questionnaires for data collection since PDAs are not conventionally adopted for data collection in emergencies.

**Table 12:** Fur Baranga: GAM point estimates, 95% CIs, LQAS result, estimated time expenditure* and cost^† ^required for data collection, by sampling design

*Sampling design*	*GAM point estimate (95% CI)*	*GAM LQAS result*	*Team days required to collect data*	*Cost for data collection*
33 × 6 design	8.1 (3.6, 12.5)	Fail to reject H_o _at 10% GAM threshold. Conclude area requires intervention appropriate for 10% threshold level	8.25	$1232
67 × 3 design	6.5 (3.0, 9.9)	Reject H_o _at 10% GAM threshold. Conclude area has <10% GAM prevalence	11.17	$1630
30 × 30 design	8.6 (6.5, 10.8)	NA	30.00	$4606

*Time expenditure results for Fur Baranga assume 8.5 hour work day.

^†^Estimated cost based on conversion rate of 237 Sudanese Dinars to 1 US Dollar.

**Table 13:** Habila: GAM point estimates, 95% CIs, LQAS result and estimated time expenditure* for data collection, by sampling design

*Sampling design*	*GAM point estimate (95% CI)*	*GAM LQAS result*	*Team days required to collect data*	*Cost for data collection*
33 × 6 Design	4.7 (1.9, 7.4)	Reject H_o _at 10% GAM threshold. Conclude area has <10% GAM prevalence	4.13	NA
67 × 3 Design	8.0 (4.5, 11.5)	Fail to reject H_o _at 10% GAM threshold. Conclude area requires intervention appropriate for 10% threshold level	5.58	NA
30 × 30 Design	6.9 (4.8, 9.0)	NA	15.00	NA

*Time expenditure results for Habila assume 12 hour work day.

Because we expect the time required for data collection would vary according to the size of the assessment area, we conducted a sensitivity analysis to assess the difference in the time required for data collection when greater distances of travel to and between clusters were assumed. Using the same formula as applied for the above time estimations, we estimated the number of team days required to complete data collection for each design, once assuming the distance of travel to and between clusters was increased by three times, and once assuming the distance of travel was increased by five times the actual average distances in Fur Baranga and Habila. Here again we allowed the length of the work day to be determined by the average length of time required to complete one (or two) 30 × 30 cluster(s) in one work day. As a result, the time estimations may under-represent the time required to complete data collection for the 30 × 30 design. Nevertheless, in all scenarios tested, the 33 × 6 and 67 × 3 designs are still estimated to require less time for data collection than the 30 × 30 design (table 14).

**Table 14:** Estimated time expenditure required for data collection, by sampling design and AU, for actual distance, 3 × actual distance, and 5 × actual distance to and between clusters

*Administrative unit*	*Distance assumption*	*Sampling design*			*Length of work day assumption*
		*33 × 6 design (in team days)*	*67 × 3 design (in team days)*	*30 × 30 design (in team days)*	

Fur Baranga	Actual distance	8.25	11.17	30.00	8.5 hr work day
Habila	Actual distance	4.13	5.58	15.00	12.0 hr work day
Fur Baranga	3× Actual distance	11.00	16.75	30.00	9.5 hr work day
Habila	3× Actual distance	8.25	13.40	30.00	9.0 hr work day
Fur Barnaga	5× Actual distance	11.00	22.33	30.00	11.0 hr work day
Habila	5× Actual distance	11.00	22.33	30.00	9.5 hr work day

## 3. Discussion

This study advances what is known about the performance of the 33 × 6 and 67 × 3 designs relative to the 30 × 30 cluster design conventionally used in emergency settings. In contrast to the earlier study by Deitchler *et al *[3], in this study we use fully independent samples representative of the same area to compare the performance of the designs. In addition, household- as well as child-level indicators were tested. Time expenditure estimates for two new assessment areas were obtained, and, for the first time, the cost of data collection by design was estimated.

Conventional sampling theory would suggest that for the same sample size, a design with more clusters and fewer observations per cluster will provide a more precise estimate for an indicator (i.e. narrower confidence interval). In comparison to the 30 × 30 design, the 33 × 6 and 67 × 3 designs collect data from more clusters and sample fewer observations per cluster. The 33 × 6 and 67 × 3 designs do not, however, have a sample size equal to that of the 30 × 30 design. The results of this study therefore provide a unique opportunity to evaluate the empirical trade-offs of each of the sampling designs with respect to the precision of estimates, and the time and cost required for data collection.

The design effect for an indicator can often be approximated using the below formula:

Deff = 1 + *p *(b-1),

where *p *is the intra-cluster correlation (rho) and b is the number of observations per cluster [19]. Assuming the intra-cluster correlation for an indicator is relatively constant among designs, we would then expect the design effect of the 30 × 30 design to be larger than that of the 33 × 6 or 67 × 3 designs for the same indicator. The empirical data from Fur Baranga and Habila conform to these expectations: 1) Intra-cluster correlations are similar among the designs, showing a difference of <0.15 for most indicators, and 2) the 30 × 30 design has the largest design effect for all indicators – the only exceptions being stunting in Fur Baranga and the prevalence of low middle upper arm circumference (MUAC) in Habila (Tables 15, 16).

**Table 15:** Fur Baranga: design effects and intra-cluster correlations (rho) for child- and household-level indicators, by sampling design

	*Sampling design*		
	*33 × 6 design*	*67 × 3 design*	*30 × 30 design*

	*Design effect (rho)*	*Design effect (rho)*	*Design effect (rho)*

*Child-level Indicators*			

GAM	1.2339 (0.0426)	0.9602 (0.0000)	1.2626 (0.0082)
Low MUAC (<12.5 cm)	1.3169 (0.0567)	0.9108 (0.0000)	1.6112 (0.0197)
Stunting	1.6427 (0.1202)	1.0429 (0.0156)	0.5487 (0.0000)
Underweight	1.2382 (0.0414)	0.8487 (0.0000)	1.2919 (0.0087)
BCG vaccination	1.8279 (0.1564)	1.4842 (0.2209)	4.0857 (0.1020)
Measles vaccination	2.1131 (0.2611)	1.7224 (0.3648)	7.4488 (0.2562)
VAC supplementation (5.5 month recall)	2.5254 (0.3087)	1.9328 (0.5023)	16.4399 (0.5503)
Diarrhea (2 week recall)	0.9708 (0.0000)	1.1977 (0.0462)	2.7355 (0.0584)
ARI (2 week recall)	2.1629 (0.2214)	0.9851 (0.0000)	3.9733 (0.1006)
Fever (2 week recall)	1.4590 (0.0850)	1.3081 (0.1497)	1.9390 (0.0305)

*Household-level Indicators*			

Access to potable water	4.4337 (0.6643)	2.6278 (0.8008)	21.3793 (0.6802)
Access to latrine	3.2750 (0.4384)	1.6980 (0.3406)	10.2704 (0.3085)
Ownership of bed net	2.1713 (0.2231)	1.3621 (0.1801)	2.3255 (0.0442)
Food shortage (5.5 month recall)	2.8305 (0.3527)	1.6889 (0.3431)	7.9581 (0.2316)

**Table 16:** Habila: design effects and intra cluster correlations (rho) for child- and household-level indicators, by sampling design

	*Sampling design*		
	*33 × 6 design*	*67 × 3 design*	*30 × 30 design*

	*Design effect (rho)*	*Design effect (rho)*	*Design effect (rho)*

*Child-level indicators*			

GAM	0.8000 (0.0000)	0.8270 (0.0000)	1.4208 (0.0129)
Low MUAC (<12.5 cm)	1.3447 (0.0621)	0.9479 (0.0000)	1.2828 (0.0083)
Stunting	1.1454 (0.0248)	0.9506 (0.0000)	1.3622 (0.0111)
Underweight	1.0007 (0.0000)	1.0523 (0.0232)	1.2840 (0.0084)
BCG vaccination	2.5743 (0.3024)	1.5682 (0.2763)	4.9646 (0.1312)
Measles vaccination	3.1927 (0.4966)	1.8578 (0.4771)	7.9908 (0.2610)
VAC supplementation (5.5 month recall)	3.4622 (0.5364)	2.1763 (0.6419)	12.9310 (0.4249)
Diarrhea (2 week recall)	1.5103 (0.0944)	1.2213 (0.1107)	4.1322 (0.1037)
ARI (2 week recall)	1.4232 (0.0774)	1.3977 (0.1985)	1.5472 (0.0171)
Fever (2 week recall)	1.4139 (0.0756)	1.1678 (0.0517)	2.1121 (0.0360)

*Household-level indicators*			

Access to potable water	5.7618 (0.9231)	2.9092 (0.9401)	27.6205 (0.8881)
Access to latrine	3.2540 (0.4344)	1.9448 (0.4627)	11.3895 (0.3456)
Ownership of bed net	3.4041 (0.4637)	2.1906 (0.5939)	12.1655 (0.3731)
Food shortage (5.5 month recall)	3.5912 (0.5000)	2.1570 (0.5772)	15.0586 (0.4688)

In terms of precision, the 30 × 30 design produces the narrowest confidence intervals for child-level indicators. The only exceptions are indicators with high intra-cluster correlations, namely, VAC supplementation and measles vaccination coverage. These results are not surprising as we would expect these indicators to cluster according to the accessibility of health services.

Although the 33 × 6 and 67 × 3 designs produce less precise results than the 30 × 30 design for anthropometric indicators, it is noteworthy that the 33 × 6 and 67 × 3 designs provide the opportunity to use a LQAS hypothesis test to assess the prevalence of GAM whereas the 30 × 30 design does not. The LQAS analysis method is particularly useful in cases where the CI for GAM overlaps with a critical threshold prevalence used for decision making. In Fur Baranga, the 33 × 6 design produced an estimate of 8.1% with a 95% CI of 3.6–12.5%. In Habila, the 67 × 3 design produced an estimate is 8.0% with a 95% CI of 4.5–11.5%. In each of these cases, it is not possible to determine from the CI alone whether the 10% GAM threshold has been exceeded or not. By using a LQAS hypothesis test, a probability-based decision can be made about the threshold prevalence. It is precisely in this way that the LQAS hypothesis test can add value to a GAM point estimate and CI, providing useful information for triangulating and interpreting data for decision making about GAM thresholds.

The intra-cluster correlation coefficients for the household-level indicators tested in this study are high (often >0.30), rendering the 30 × 30 sampling design not only inefficient (design effects >10.0), but also imprecise. The 67 × 3 design, in contrast, maintains low design effects (in spite of intra-cluster correlations >0.30) and produces more precise results. Even the 33 × 6 design provides results nearly as precise as the 30 × 30 design; the 700 extra observations required for the 30 × 30 sample size offer little advantage to the estimation of household-level indicators such as those tested here.

In terms of time expenditure, the 33 × 6 and 67 × 3 designs offer a clear benefit over the 30 × 30 design. In Fur Baranga and Habila, estimates for all indicators could have been obtained between one-quarter and one-third of the time with the 33 × 6 and 67 × 3 designs, and at cost savings of similar magnitudes. Contrary to our expectations, in this field application, data collection for the 67 × 3 design required less total driving (in km) than the 30 × 30 design (data only available for Fur Baranga). In Fur Baranga, data collection for the 33 × 6 design required 95 total km of driving, the 67 × 3 design required 231 km and the 30 × 30 design required 244 total km. Although somewhat surprising, these results can be explained. Our experience has shown that travel for the 33 × 6 and 67 × 3 designs can be planned strategically so that one team might be able to complete all of the clusters located in a far away region in one day. This is not as feasible with the 30 × 30 design because a team cannot usually complete more than one cluster per day if travel to the region is of substantial distance. Moreover, in cases where the sampling frame for an assessment area is relatively small (e.g. <50 primary sampling units listed), many of the areas listed in the sampling frame will be selected by proportional-to-population-size sampling for multiple clusters of the 67 × 3 design, which can also reduce the amount of total travel necessary. These potential advantages are expected to be common to most applications of the 33 × 6 and 67 × 3 designs; however, the time and cost savings reported here are valid only for the Fur Baranga and Habila areas assessed in this study.

We expect the time and cost required for data collection would vary by design according to a number of factors, including, the size of the area being assessed, road infrastructure, dispersion of households, and the security situation. Because the geographic size of the AUs assessed for this field validation were small (e.g. the furthest distance to any cluster was 30 km in Fur Baranga), travel time and cost of transport for each design remained limited. We believe the 33 × 6 design would provide a time and cost advantage over the 30 × 30 design in almost any circumstance. The 67 × 3 design also has the potential for wide applicability, as indicated by our sensitivity analysis, but may provide the most time and cost benefit for sampling smaller areas when detailed information is needed to prioritize the areas most affected.

In the absence of a gold standard (as would be provided by census data), it is not possible to determine which design (33 × 6, 67 × 3, or 30 × 30 cluster survey) produced the most accurate results in this study. However, the overlap of the CIs across designs does provide some evidence for the comparability of results obtained for the indicators tested, the main trade-off of the 33 × 6 and 67 × 3 designs being the wider CIs produced for child-level indicators, compared to the 30 × 30 design. We believe this is acceptable in view of the time and cost savings over the 30 × 30 design, and the added benefit of LQAS hypothesis testing for key thresholds of GAM prevalence.

Tentatively, the variable results obtained in Fur Baranga for the food shortage indicator may be explained by the more subjective nature of the indicator, which is based on self-report of a food shortage since a local event five months preceding the interview. Answers for this type of indicator may be less consistent than for anthropometry or vaccination indicators which are collected using more standardized methods. Similarly, we expect the results for the ARI indicator in Fur Baranga may be due to the difficulty of caregiver diagnosis of the symptoms associated with ARI.

Our results suggest that the sample sizes of the 33 × 6 and 67 × 3 designs are too small to provide precise and usable epidemiologic measures of a rare event such as mortality. This limitation, however, may not be unique to the 33 × 6 and 67 × 3 designs. The utility of a 30 × 30 cluster survey for deriving estimates of the crude and under five mortality rates is the subject of debate. Some studies have shown 30 × 30 cluster surveys to provide mortality estimates with limited precision, the CIs overlapping multiple thresholds of interest, and, in conflict situations, design effects as high as eleven or more have been documented [2,20-23]. Although the 30 × 30 design is commonly used for assessment of retrospective mortality, it has not, to our knowledge, been validated for this purpose [2].

One issue of concern is the divergent results noted among 33 × 6 and 67 × 3 designs within each AU for the 10% GAM threshold. Aside from the increased alpha and beta errors due to the reduced sample size of the designs, the concepts of lower and upper thresholds also help discuss the issue. In this LQAS application, the populations most in need (i.e. where the true GAM prevalence is ≥ upper threshold) and the populations least in need (i.e. where the true GAM prevalence is ≤ lower threshold) are correctly categorized within the stated alpha and beta error limits. Populations with a true GAM prevalence falling in-between those upper and lower thresholds are, however, categorized with higher error [6]. For example, if the true prevalence of GAM is between the respective upper and lower thresholds of 10% and 5%, we would then expect an increased likelihood (beyond the defined tolerable beta error) for misclassification at the 10% GAM threshold. That being said, in the case of GAM prevalence, it is less critical to make a type II error than it is to make a type I error. Sampling designs such as this, which minimize the risk of misclassifying areas with a true prevalence above a critical threshold level, are particularly relevant in emergency settings, when lives can depend on the timely detection of areas requiring humanitarian aid.

Issues related to statistical error and precision are of concern for all population-based surveys. These technical issues should always be considered in the context of the survey's purpose. In emergency settings, they should be balanced against the urgency of the situation and the extent of resources available for data collection and humanitarian response. While the level of precision offered by the 33 × 6 and 67 × 3 designs for child-level indicators may not be appropriate for the purpose of every survey, this study demonstrates that the 33 × 6 and 67 × 3 designs can fulfill rapid data collection needs in an emergency and volatile security situation, while still providing results of sufficient precision to allow for the identification of priorities and initiation of timely humanitarian response. The wider CIs of the 33 × 6 and 67 × 3 designs for anthropometric indicators are counter balanced by the opportunity to conduct a LQAS hypothesis test to detect whether or not a critical threshold of GAM prevalence has been exceeded in the assessment area. The 33 × 6 and 67 × 3 designs provide reliable and necessary information at a fraction of the time and cost required by the conventional 30 × 30 sampling design, allowing for a savings of not only time and resources, but one hopes, of lives as well.

## List of abbreviatons

AED: Academy for Educational Development; ARI: Acute Respiratory Infection; BCG: Bacille Calmette Guerin; AU: Administrative Unit; CI: Confidence Interval; CRS: Catholic Relief Services; Deff Design Effect; FANTA: Food and Nutrition Technical Assistance; GAM: Global Acute Malnutrition; GLLAMM: Generalized Linear Latent and Mixed Models; LQAS: Lot Quality Assurance Sampling; MUAC: Middle Upper Arm Circumference; NCHS: National Center for Health Statistics; OSU: Ohio State University; PDA: Personal Digital Assistant; PPS: Probability Proportionate to Size; PSU: Primary Sampling Unit; SC: Save the Children; SMART: Standardized Monitoring and Assessment of Relief and Transitions; SRS: Simple Random Sample; USAID: United States Agency for International Development; VAC: Vitamin A Capsule; WHO: World Health Organization.

## Competing interests

The authors declare that they have no competing interests.

## Authors' contributions

MD, HD, and GB designed the study. MD and HD led the collection and analysis of data for this study. MD, HD, and GB all participated in the writing of the manuscript. All authors read and approved the final version of the manuscript.
